# The road from manual to automatic semantic indexing of biomedical literature: a 10 years journey

**DOI:** 10.3389/frma.2023.1250930

**Published:** 2023-09-29

**Authors:** Anastasia Krithara, James G. Mork, Anastasios Nentidis, Georgios Paliouras

**Affiliations:** ^1^Institute of Informatics and Telecommunications, National Center for Scientific Research “Demokritos”, Athens, Greece; ^2^National Library of Medicine, Bethesda, MD, United States; ^3^Department of Informatics, Aristotle University of Thessaloniki, Thessaloniki, Greece

**Keywords:** biomedical information, semantic indexing, BioASQ challenge, biomedical literature, NLM

## Abstract

Biomedical experts are facing challenges in keeping up with the vast amount of biomedical knowledge published daily. With millions of citations added to databases like MEDLINE/PubMed each year, efficiently accessing relevant information becomes crucial. Traditional term-based searches may lead to irrelevant or missed documents due to homonyms, synonyms, abbreviations, or term mismatch. To address this, semantic search approaches employing predefined concepts with associated synonyms and relations have been used to expand query terms and improve information retrieval. The National Library of Medicine (NLM) plays a significant role in this area, indexing citations in the MEDLINE database with topic descriptors from the Medical Subject Headings (MeSH) thesaurus, enabling advanced semantic search strategies to retrieve relevant citations, despite synonymy, and polysemy of biomedical terms. Over time, advancements in semantic indexing have been made, with Machine Learning facilitating the transition from manual to automatic semantic indexing in the biomedical literature. The paper highlights the journey of this transition, starting with manual semantic indexing and the initial efforts toward automatic indexing. The BioASQ challenge has served as a catalyst in revolutionizing the domain of semantic indexing, further pushing the boundaries of efficient knowledge retrieval in the biomedical field.

## 1. Introduction

A vast amount of biomedical knowledge is published every day in the literature and in structured resources like biomedical ontologies. It is a challenge for biomedical experts to identify and process all the available knowledge. For example, 1.3 million citations were added to MEDLINE/PubMed during 2018,^1^ which corresponds to more than two citations per minute. In this context, the identification of articles relevant to a specific research topic is very hard. Efficient access to relevant knowledge is crucial and simple term-based search can retrieve irrelevant documents (e.g., due to homonyms) or miss relevant documents (e.g., due to synonyms, abbreviations, or term mismatch). Much effort has been made to address this issue, including semantic search approaches that use predefined concepts which come with several associated synonyms and relations to other concepts. The goal of semantic indexing is to use this semantic information to improve the quality of information retrieval, e.g., through query expansion. Toward this direction, the National Library of Medicine (NLM) indexes citations in the MEDLINE database with topic descriptors from the Medical Subject Headings (MeSH) thesaurus. This semantic indexing process allows MEDLINE/PubMed to offer advanced semantic search strategies, that can retrieve citations relevant to a topic of interest, addressing issues such as synonymy and polysemy of biomedical terms.

During the last years important advancements have been achieved in the area of semantic indexing. In particular, the use of Machine Learning has helped to move gradually from manual to automatic semantic indexing in the biomedical literature. Since 2012, BioASQ has initiated a shared task for semantic indexing, allowing leading teams in the field to advance their approaches and improve significantly their performance. This performance improvement has led to the adoption of fully automated indexing by NLM since 2022. In this paper, the road of this transition is presented: how manual semantic indexing has started, the first efforts toward automatic indexing, and how the BioASQ challenge has helped in the revolution of the domain.

## 2. Manual semantic indexing

### 2.1. How it all started

**Figure 1:**
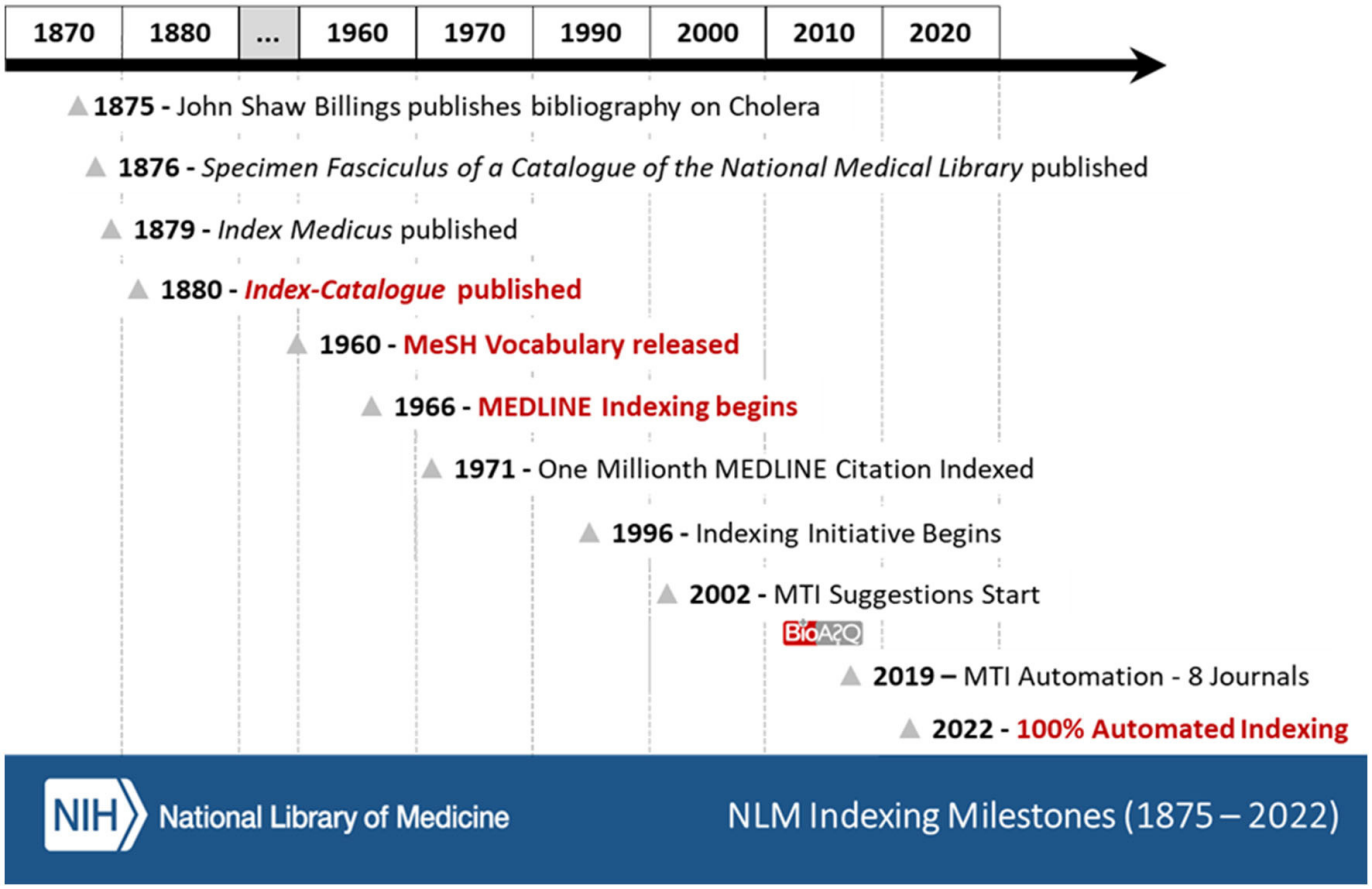
NLM indexing milestones (1875–2022). The red lettered milestones highlight important turning points in the maturity of MEDLINE^Ⓡ^ indexing at NLM.

There are several very good references on the history of the US National Library of Medicine (Miles, 1982; Blake et al., 1986; Reznick and Koyle, 2017; NLM, 2023a) and one of these, *A History of the National Library of Medicine: The Nation's Treasury of Medical Knowledge by* Miles (1982) goes into depth on how indexing began and evolved. Figure 1 details important milestones selected from these various references with regards to how indexing at NLM started, matured, and evolved over time.

History tells us (Miles, 1982; NLM, 2023a) that the impetus for developing a catalog and index of the medical literature may have started as early as 1859 when John Shaw Billings was preparing his thesis on the surgical treatment of epilepsy. A great deal of time and effort was spent by Billings going through the medical literature looking for what he needed. In 1874, Billings began preparing the first index of the medical literature with the goal of making it easier for him and others to find topics in the medical literature. This drive to index the medical literature, provide information freely to others, and expand access to the medical literature is still part of NLM's Mission Statement: “its mission of enabling biomedical research, supporting healthcare and public health, and promoting healthy behavior” (NLM, 2020). Billings enlisted fellow military medical officers to spread the indexing effort across more people and to index medical literature that he did not have access to Miles (1982). To ensure uniformity, Billings provided detailed guidance on what he wanted done and how it should be formatted (Miles, 1982).

Following the cholera epidemic of 1873 in the United States, Billings was asked to put together a bibliography on all available cholera literature of the day as part of an overall cholera review that was being undertaken by the Army. In 1875, Billings published his cholera bibliography. This bibliography on cholera provided the first test of Billings' indexing methodology and proved to Congress and the medical community how useful an index of this kind could be and was a precursor to the Index Medicus (NLM, 2020). In 1876, Specimen Fasciculus of a Catalogue of the National Medical Library (US Library of the Surgeon-General's Office, 1876) was published, containing 72 pages of indexing covering Aabec to Air and was sent to members of Congress and prominent people in the medical community and was well-received (Miles, 1982). In 1879, Index Medicus was published (Index Medicus, 1879) and in 1880, Index-Catalogue was published (Greenberg and Gallagher, 2009). The Index-Catalogue was only published in full every 4 years, so during the interim years, the companion Index Medicus was published with the preceding year's updates. Both publications were instrumental in setting the stage for what later became the U.S. National Library of Medicine, how biomedical indexing would be handled at NLM, and drive NLM's mission to disseminate biomedical information to as wide an audience as possible. The Index Medicus continued being published until 2004 (NLM, 2022b).

In 1960, the MeSH Controlled Vocabulary was published (NLM, 2010) providing indexers for the first time with a limited set of 4,300 terms to be used when indexing the literature. The controlled vocabulary provided for more consistency of the final indexing and made searching for information easier. MeSH continues to be updated and expanded as necessary as new information or better understanding of the existing information becomes available. The 2022 MeSH Vocabulary (NLM, 2023c) now contains more than 30,000 Descriptors, over 300,000 Supplementary Concept Records (SCRs), and 76 Qualifiers (also known as Subheadings).

By 1966, what we would today recognize as NLM MEDLINE^Ⓡ^ indexing began (NLM, 2022b). MEDLINE indexing began with new data entry and indexing standards with the goals of having data indexed by humans, stored in a database, and easily retrievable by librarians (at the time) and later be accessible by the public. Indexing standards and storage requirements continue to change over time as technology improves and NLM's indexing focus evolves and changes to keep up with the biomedical field.

In 1971, the One Millionth MEDLINE article was indexed.^2^ The exact date and PMID for the one millionth indexed article is not identified in the various references, but a quick search in PubMed/MEDLINE provides a close approximation of it likely happening toward the end of January or the first part of February 1971. In 2022, just over 50 years later, we are closing in on the 30 millionth indexed article (PubMed Query: medline [sb]).

### 2.2. Obstacles on the way

**Figure 2:**
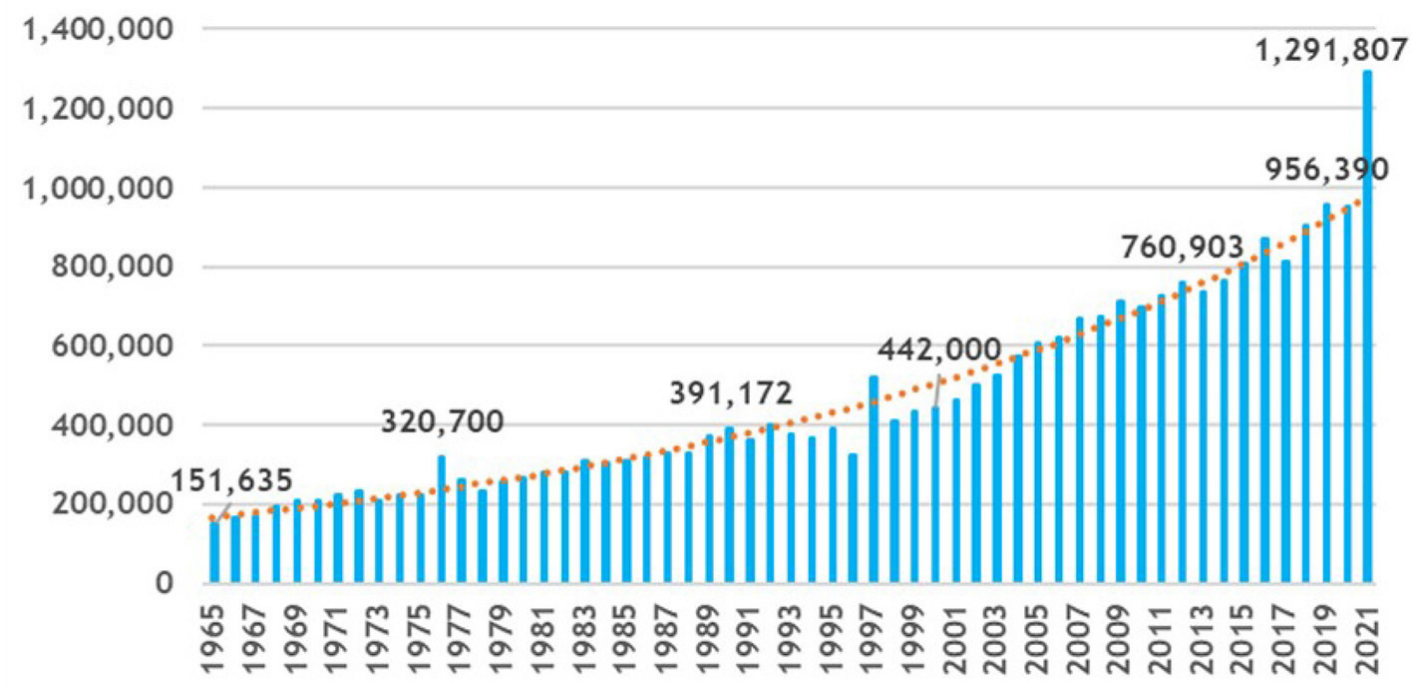
Citations added to MEDLINE^®^ by fiscal year FY65–FY21.

For nearly 150 years, the NLM has provided access to biomedical literature through the analytical efforts of human indexers. Figure 2 shows the increasing number of articles indexed by NLM from 151,635 articles in Fiscal Year (FY) 1965 to almost 1.3 million articles in FY 2021 (NLM, 2018a, 2022a,c). The exponential trend line in Figure 2 is shown in orange. During this same timeframe, the number of indexed journals increased from 2,241 in FY 1965 to 5,282 in FY 2021 (NLM, 2018a). The volume and complexity of MeSH has also continued to increase. Where the original 1960 MeSH Vocabulary contained just 4,300 Descriptors, the 2022 MeSH Vocabulary (NLM, 2023c) now contains 30,194 Descriptors. They are hierarchically organized into a directed acyclic graph with 16 branches representing specific areas of biomedical information which expands to 255,727 terms when Entry Terms are also considered. There are also 317,992 terms for Supplementary Concept Records (SCRs) which balloons to 706,836 SCRs when their Synonyms are added. 2022 MeSH also contains 76 Qualifiers (also known as Subheadings), each with rules as to what Qualifiers are allowed for each of the 30,194 Descriptors. There is also a myriad of indexing rules for when to use Descriptors, which ones are required, which ones need to be coordinated with which other Descriptors, when is it appropriate to use Qualifiers with Descriptors, etc. (NLM, 2018c).

This continual need for growth and ever-increasing complexity along with flattening budgets all contributed to a backlog of 576,735 articles in PubMed^®^ that should be indexed but have not yet been indexed (PubMed Query: inprocess[sb] on January 4, 2021) and the average time to index for articles fully indexed by humans was 145 days in FY 2021 (NLM, 2023b).

## 3. Automatic semantic indexing before BioASQ

### 3.1. How it started

The Semantic Web (Berners-Lee et al., 2001; Antoniou and van Harmelen, 2008) is an effort to establish standards and mechanisms that will allow computers to reason more easily about the semantics of the Web's resources (documents, data, services etc.), enabling them and ultimately their users to share, locate, and integrate resources more easily.

Following the popularity of the Semantic Web as a research topic, the term “semantic” is now often used to denote technologies that exploit resources with explicit, formally defined semantics. In that context, the term *semantic search engine* refers to systems that attempt to match queries to relevant information from structured data (e.g., databases, taxonomies, ontologies), or systems that aim to match queries to relevant documents or snippets by using resources with formally defined semantics as a mediator (Dong et al., 2008; Bast et al., 2016). In the latter case, queries and documents may be automatically annotated with concepts from taxonomies or ontologies to facilitate the matching of related queries and documents that use synonymous, polysemous, or semantically relevant terms, instead of (or in addition to) relying on surface string-level matching (e.g., keyword matching). The automated annotation of queries and documents with concepts from taxonomies and ontologies can be performed by relying on hierarchical classification algorithms.

Semantic search engines aim to surpass conventional search engines (i) by producing better rankings of relevant information, for example by matching queries to results at the conceptual level; (ii) by reducing redundant results, for example by aggregating results that express the same concepts; (iii) by increasing the coverage of the results, for example by expanding queries with semantically related terms; (iv) by presenting the results in a more comprehensible manner, for example by allowing the results to be grouped by the concepts of the query. In the biomedical domain, search engines such as GoPubMed (Dietze et al., 2008), HubMed (Eaton, 2006),^3^ ClusterMed,^4^ EBIMed (Rebholz-Schuhmann et al., 2007), XplorMed (Perez-Iratxeta et al., 2007) addressed specialized needs by processing biomedical literature in full text or abstracts as these become available in PubMed. These engines employed domain-specific background knowledge in the form of hierarchical thesauri, ontologies, such as disease and gene ontology, and, hence, qualified as semantic search engines. They typically exploited few of the available domain-specific resources, however, whereas in practice multiple resources of different types need to be combined. There are also commercial solutions like OVID.^5^

### 3.2. Medical Text Indexer (MTI)

Toward this direction, the US National Library of Medicine (NLM) established the Indexing Initiative project back in 1996 (Aronson et al., 1999; Mork et al., 2017). This cross-library team's mission was to explore indexing methodologies for ensuring quality and currency of NLM document collections. The NLM Medical Text Indexer (MTI) is the main product of this project.

**Figure 3:**
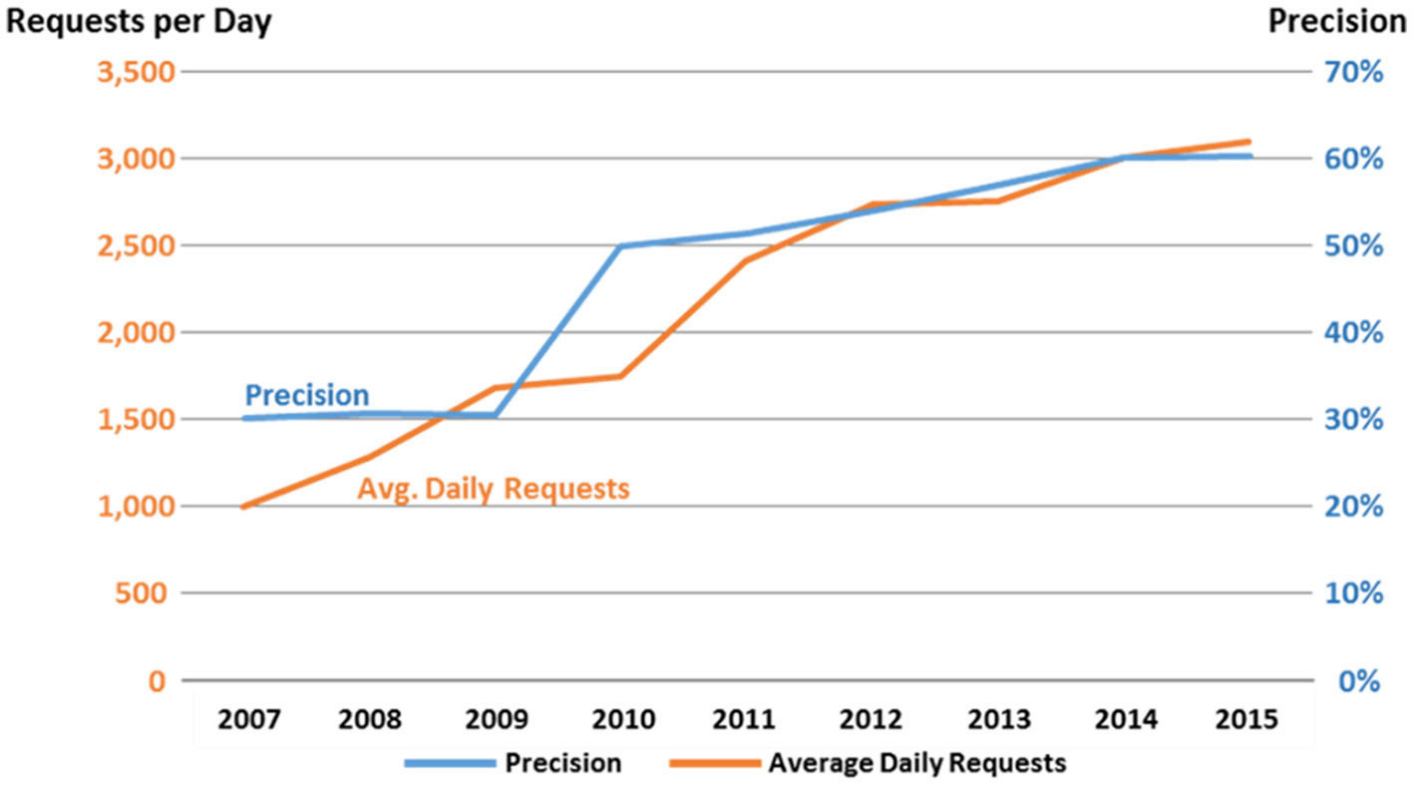
MTI precision and indexer use 2007–2015.

The NLM Medical Text Indexer (MTI) combines human NLM Index Section expertise and Natural Language Processing technology to curate the biomedical literature more efficiently and consistently. In 2002, MTI started being used to provide indexing recommendations to the NLM indexers (Mork et al., 2017). Figure 3 graphs MTI Precision and the accompanying usage of MTI by the NLM indexers. Between 2007 and 2010, Precision was a not very impressive 30%. The reason for this can be found in how MTI initially provided indexing recommendations to the indexers. The original idea was to provide a relatively long list of potential indexing recommendations to the indexers so that they could pick and choose what they wanted from the list, so MTI was heavily balanced toward Recall. What we found out from the indexers around 2010, was that this was causing them to second guess their indexing and taking them longer to index an article. The indexer wasn't sure if the terms were simply bad recommendations from MTI, or if they might have missed something in the article, so they then had to go back and review the article again. The solution to this was to rebalance MTI toward a more even Recall/Precision mix and this is easily seen in Figure 3 where the Precision goes up almost immediately from 30 to 50%. MTI continued to improve Precision through the years and as can be seen in the Figure 3 graph, indexer usage and acceptance of MTI increased along a similar trajectory.

The increased acceptance and confidence in the MTI indexing created the opportunity in 2011 for MTI to be used as a First Line Indexer (MTIFL). The indexers noticed that for a small set of journals, MTI performed very well, and these journals were the first to be processed by MTIFL. In Figure 6, you can see how much better performing MTIFL was performing over the standard MTI processing by looking at the purple line that appears in 2011. For the first time, MTI indexing would be considered the same as a novice indexer and simply revised by a more experienced indexer. This was the first of many small steps toward fully automatic indexing.

### 3.3. BioASQ challenge

Since 2012 a dedicated shared task has been organized for the automated semantic indexing of biomedical literature, in the context of the annual BioASQ challenge (Tsatsaronis et al., 2015). In the first year, BioASQ introduced two tasks, namely *Task a*, on large-scale semantic indexing of biomedical literature, and *Task b*, on biomedical semantic information retrieval, question answering, and summarization.

*Task a* in particular, was built upon the standard procedure of semantic indexing citations with topic descriptors from MeSH at NLM. In particular, the participants were provided with titles and abstracts of new biomedical articles written in English, as they became available online and prior to their annotation with MeSH labels by the MEDLINE curators. The participants were then asked to employ their approaches to automatically annotate these new articles with MeSH labels and submit their predictions to BioASQ, that is to classify them into the topic classes provided by MeSH. Later, when manual annotations became available for these articles, they were used as ground-truth annotations to evaluate the classification performance of participating systems. In order to develop their systems, the participating teams were also provided with a training dataset of older articles, where manual MeSH annotations were already available at MEDLINE. In effect, this is an extreme multi-label text classification task (XMTC), as each article belongs to only some of the several thousand classes provided by MeSH. The classes are also hierarchically organized and the systems were required to assign the narrower labels applicable to each document, as done by the MEDLINE curators.

**Figure 4:**
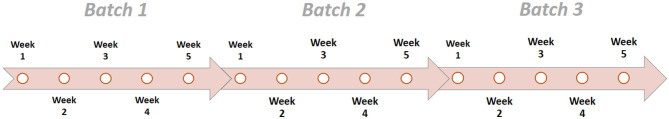
The distribution of weekly *Task a* testsets into three batches over a period of about 4 months annually.

*Task a* was structured into several weekly testsets, distributed over a period of about 4 months, February to May, annually. The testset, consisting of new, unclassified documents, was released each Monday, and the participants had about twenty-four hours to produce their predictions and submit their responses before some manual annotations become available by the MEDLINE curators. The weekly testsets were organized into three batches, running for several weeks each, in total. In particular, in the first version of *Task a*, each batch consisted of six testsets. For the remaining nine annual versions, each batch consisted of five weekly testsets as presented in Figure 4. During the course of the task, preliminary results were published for early testsets, based on any manual annotations already available, in order to let the participants know how different versions of their systems perform and introduce new ideas for the remaining testsets. The official results were calculated after the course of the task, once a sufficient proportion of documents in each testset received manual annotations by the MEDLINE curators.

Distinct winning teams were announced for each batch of *Task a*, considering the four best positions achieved by a team in any of the testsets of that batch. The classification performance of the systems participating in *Task a* was assessed with a range of evaluation measures. These include variants of standard information retrieval measures for multi-label classification problems (e.g., precision, recall, f-measure, accuracy), as well as measures that use the MeSH hierarchy to provide a more refined estimate of the systems' performance. The official measures for identifying the winners of the task were the micro-averaged F-measure (MiF) and the Lowest Common Ancestor F-measure (LCA-F; Tsoumakas et al., 2009; Kosmopoulos et al., 2015).

### 3.4. Resources

In the context of the BioASQ challenge, a training dataset was provided to the participants in order to develop their systems for *Task a*. The final version of this dataset with documents that have already been labeled by the expert indexers at NLM was produced in 2022, for the tenth version of *Task a*. This training dataset consists of 16,218,838 articles, from the PubMed Annual Baseline Repository for 2022, annotated with 12.68 MeSH labels per article, on average. In total, the dataset covers 29,681 distinct MeSH labels out of the 30,213 descriptors available in MeSH 2022.

During the course of the tenth version of *Task a*, several weekly testsets were provided to the participants, each consisting of some thousands of unlabeled articles as presented in Table 1. Eventually, after the participants submitted their responses, most of these articles received manual labels by the NLM indexers, as shown in Table 1, which were used for the evaluation of the predictive performance of participating systems.

**Table 1:** Statistics on test datasets for the tenth version of *Task a*.

**Batch**	**Articles**	**Annotated articles**	**Labels per article**
1	9,659	9,450	13.03
	4,531	4,512	12.00
	4,291	4,269	13.04
	4,256	4,192	12.81
	4,862	4,802	12.75
Total	27,599	27,225	12.72
2	8,874	8,818	12.70
	4,071	3,858	12.38
	4,108	4,049	12.60
	3,193	3,045	11.74
	3,078	2,916	12.07
Total	23,324	22,686	12.29
3	2,376	1,870	12.31
	28	0	−
Total	2,404	1,870	12.31

Due to the early adoption of a new NLM policy for fully automated indexing, the third batch finally consists of a single test set.

### 3.5. Evolution of approaches

A variety of alternative approaches have been proposed for the automated semantic indexing of biomedical literature, in the context of the BioASQ challenge, during the last 10 years.

During the first years of BioASQ, most approaches focused on traditional methods, both in terms of representation as well as in concept matching. In particular, during the early years, bag-of-words and TF-IDF representations were proposed (Balikas et al., 2015), in contrast to most recent years, where the approaches focus on neural word and paragraph embeddings (Nentidis et al., 2022). For finding the relevant MeSH labels, the trend moved from traditional machine learning approaches, such as KNN, SVM, and Learning-to-Rank (Balikas et al., 2015), toward Deep Networks and Attention Mechanisms (Nentidis et al., 2022).

As a result, the most recent dominant approaches in this task, were based on Deep Learning (DL) architectures. For example, *BERTMeSH* by You et al. (2021) succeeded their previous approach on label tree-based DL method of *AttentionXML* (You et al., 2018). The latter came with two unique features: (1) a multi-label attention mechanism with raw text as input, which allows to capture the most relevant part of text to each label; and (2) a shallow and wide probabilistic label tree (PLT), which allows to handle millions of labels. BERTMeSH has two technologies: (i) the state-of-the-art pretrained deep contextual representation, Bidirectional Encoder Representations from Transformers (BERT), which makes BERTMeSH capture deep semantics of full text. (ii) A transfer learning strategy focusing on both full text in PubMed Central (PMC) and title and abstract in MEDLINE, to take advantage of both. In the same direction, the methods proposed by the NLM team (Rae et al., 2021) rely on recommendations from a Convolutional Neural Network (CNN) which are ranked by a pre-trained transformer model (PubMedBERT) fine-tuned on this task. Finally, the very successful approach “*dmiip_fdu*” by the Fudan University team was based on a Learning-to-Rank approach, where the component methods include both the above-mentioned deep-learning-based BERTMeSH, as well as traditional SVM-based methods.

The most recent participating systems along with their corresponding approaches are listed in Table 2.

**Table 2:** Examples of systems and approaches for task 10a.

**System**	**Approach**
BERTMesh	pecos, tf-idf, linear model, BertMesh, PubMedBERT, multilabel attention head
NLM	SentencePiece, CNN, embeddings, ensembles, PubMedBERT
dmiip_fdu	BertMesh, PubMedBERT, BioBERT, LTR, SVM

### 3.6. Pushing systems performance

**Figure 5:**
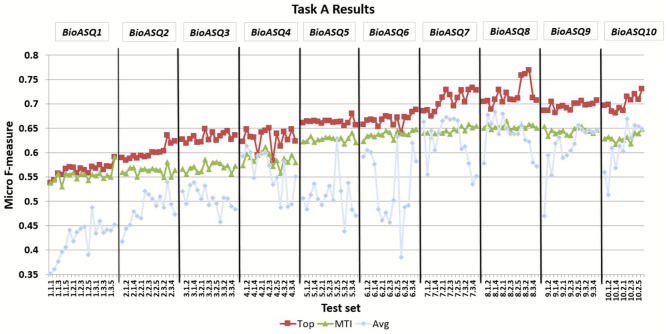
The micro f-measure (MiF) achieved by systems across different years of the BioASQ challenge. For each test set the MiF score is presented for the best performing system (Top) and the MTI, as well as the average micro f-measure of all the participating systems (Avg).

BioASQ offered the opportunity to the respective research community to compete against each other as well as against strong baselines, and hence helped to push the performance of the participating systems significantly. Figure 5 presents the improvement of the MiF scores achieved by both the MTI baseline and the top performing participant systems through the 10 years of the BioASQ challenge. The results of the task reveal that several participating systems manage to outperform the strong baselines in all test batches, considering either the flat or the hierarchical measures. In particular, the best systems have improved by more than 15 points during the 10 years. During BioASQ 8 and 9, the system performance was almost stable with minor improvement. The reason for this is the COVID-19 pandemic, which has dominated the biomedical literature during these 2 years, and has changed the distribution of the literature on various topics. The development of deep neural networks for natural language processing in the general domain during the last years has contributed to the improvement of automatic indexing. BioASQ allowed to channel this profuse methodological development into biomedical semantic indexing. Although a slight trend toward improved scores can be still observed in the results of the tenth year, the task seems to have successfully completed its main goal, concluding its life cycle.

### 3.7. Gradual improvement of semantic indexing in NLM

**Figure 6:**
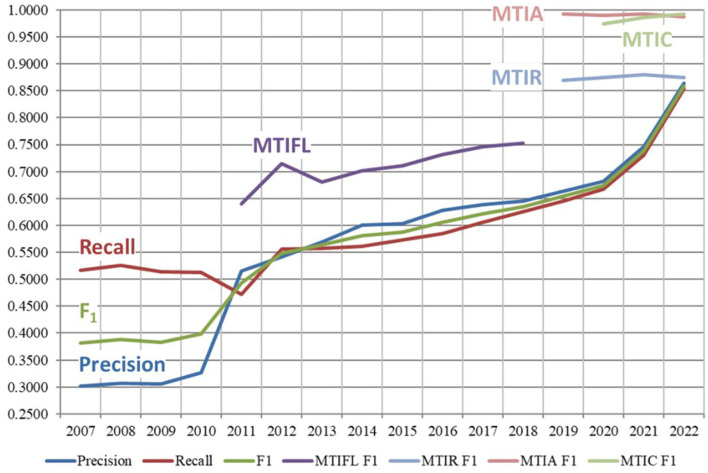
Performance of MTI and variants of it from 2007 to 2022. MTIFL, MTI First Line indexer; MTIR, MTI Review; MTIA, MTI Automatic; MTIC, MTI Comment On.

Figure 6 shows the overall performance of MTI and different variants of it from 2007 to 2022. Performance data beyond the switchover in April 2022 to fully automatic indexing is not comparable, so not included here. Recall is shown in red and goes from 0.5163 to 0.8541, Precision is shown in blue and increases from 0.3019 to 0.8646, F1 is shown in green and goes from 0.3810 to 0.8593.

The different variants of MTI indicate the methodical progression toward fully automatic indexing. MTI First Line Indexer (MTIFL) was in use from 2011 to 2020 and is shown in purple with F1 going from 0.6399 to 0.7712. The MTIFL variant started when indexers noticed that MTI performed very well on a small subset of journals (originally only 14 journals). For the first time, MTI was treated the same as a junior indexer where the MTI indexing was reviewed by a more senior indexer. MTI Comment On (MTIC) started in 2017 but statistics were not tracked until 2020. It is shown in light green with F1 0.9742–0.9931. MTIC included the title of the article being commented on to enrich the usually terse text of articles commenting on another article. MTIC replaced the practice of just copying over the indexing from the originating article. MTI Review (MTIR) was in use from 2017 to 2022 and is shown in light blue with F1 of 0.8874–0.8752. MTIR was the staging area to see how journals performed using the MTI Automatic indexing algorithm (described next). Indexers would review every article indexed by MTIR only using the title and abstract (as opposed to the full text of an article which they normally indexed from) to evaluate the MTIR indexing. This provided a realistic review of how the automatic indexing would perform for each journal. Finally, MTI Automatic (MTIA) started in late 2019 and is shown in light red with F1 going from 0.9924 to 0.9871. MTIA is like MTIR except that for MTIA, there is no complete indexer review of the articles. For MTIA, indexers only review a small sample of the articles to ensure quality and completeness of the indexing. MTIR provided the most accurate measure of how well MTI performed since the vast majority of MTIA results are not manually reviewed. This small sampling of the MTIA results likely contributes to the false boost in performance of MTIA over MTIR that we see in Figure 6. MTIA and MTIC are still being used until transition is complete to the next generation of MTI which is discussed in Section 4.2.

In 2013, the first BioASQ Challenge—Large-scale Online Biomedical Semantic Indexing (Ngomo and Paliouras, 2013) took place. For the first time the MTI development team knew of and was able to collaborate with researchers from around the world all focused on the same task of Biomedical Semantic Indexing. The Challenge provided a mechanism to highlight MTI on an international stage and more importantly, opened the window to research being done or initiated due to the Challenge in this domain. This research from the BioASQ Semantic Indexing Challenge has helped improve MTI over its entire 10-year span. Two major changes made to MTI that were inspired by the work presented at the challenges are Vocabulary Density (Mork et al., 2014) and Learning to Rank (Mao and Lu, 2013; Zavorin et al., 2016), which we present below.

The potential for MTI improvement using journal-specific data was discussed by Tsoumakas et al. (2013) during the first BioASQ workshop. In MTI, this approach was called Vocabulary Density (Mork et al., 2014) based on our findings that on average, only 999 unique MeSH Headings of the 27,149 available in 2014 MeSH were used per journal in the 6,606 journals in our Corpus. 83.81% of the used MeSH Headings were found in 500 or fewer journals and 271 MeSH Headings were only found in a single journal. This tendency of the MeSH vocabulary to be centered around specific journals allowed us to develop rules for adding or removing MeSH Descriptors based on their recent past performance. Each year we update the Vocabulary Density information by looking at how frequently each MeSH Descriptor has been used by each journal over the previous 5 years. Limiting the timeframe to 5 years keeps the information current and in line with indexing policy. An example of how we use this is for the journal Cryobiology (0006252), we see that over the last 5 years, not surprisingly, the MeSH Descriptor Cryopreservation was indexed for 88.95% of articles, so MTI can with fairly high confidence, suggest this term even if there is no indication that it would be appropriate in the Title and Abstract. Implementing this simple approach led to a statistically significant 4.44 percentage points improvement in Precision.

The task of MEDLINE semantic indexing can be formulated as a ranking problem: given a new citation, can we find those MeSH Headings that are the most relevant to this citation? In the first BioASQ Workshop, Mao and Lu (2013) discussed how they were able to apply the Learning-to-Rank (Mao and Lu, 2013; Zavorin et al., 2016) methodology to biomedical indexing. Learning-to-Rank was used to improve MTI performance by reevaluating the final list of recommendations being made by MTI to help move more relevant terms closer to the top of the list and less relevant terms lower down in the list. Two classes of MeSH Descriptors saw a significant performance boost using Learning-to-Rank. Historical Checktags (e.g., History, Eighteenth Century) and “as Topic” Descriptors (e.g., Randomized Controlled Trials as Topic). In both cases, prior to Learning-to-Rank, MTI performed so poorly that the terms were not suggested. With the Learning-to-Rank methodology added to MTI, there was significant improvement from not recommending any of these terms, to 86.49% Precision for Historical Checktags, and 69.56% Precision for “as Topic” terms.

As mentioned above, through the years MTI incorporated some of the advancements discussed in the yearly BioASQ workshops. Not all advancements were incorporated into MTI mainly due to limited resources of MTI, but also due to the added complexity of MTI. While the BioASQ Challenge focused on indexing MeSH Descriptors, MTI was also indexing MeSH Subheadings, Supplementary Concept Records, and Publication Types so all changes to MTI had to be carefully added.

## 4. The road ahead

### 4.1. Fully automatic semantic indexing is here

NLM moved to fully automatic indexing in early April 2022^6^ (NLM, 2021). As mentioned in Section 3.7, by January 4, 2021, there was an indexing backlog of 576,735 articles and an average time to index of 145 days. NLM was able to eliminate the backlog and reduce the overall time to index an article to <24 h with the move to fully automatic indexing.

Fully automatic indexing has been in the works for several years as NLM gradually improved and expanded the MTI algorithm and validated automatic indexing. In the fall of 2015, NLM automatically re-indexed all 2,011,000 OLDMEDLINE records to add additional MeSH Headings and complete a project that mapped OLDMEDLINE Other Term (OT) subject headings to MeSH Headings (NLM, 2015). In 2017, the automatic indexing of Comment On articles (MTIC) began, as mentioned in Section 3.6. In 2018, NLM added a new attribute to the XML MedlineCitation tag “ < MedlineCitation Status = “MEDLINE” IndexingMethod = “Automated” Owner = “NLM” >” identifying whether an article had been indexed without MTI support (empty attribute), Curated MTI results by a human indexer (Curated), or fully automatic MTI indexing (Automated) for all articles in MEDLINE (NLM, 2018b). The updated XML tag allowed researchers to selectively use or ignore articles in MEDLINE that were fully and/or partially automatically indexed. In late 2019, NLM started fully automatic indexing (MTIA) for eight journals as a pilot project. The success of the pilot allowed NLM to expand the effort to include automatically indexing 40% of the journals in 2021, and then to move to 100% of the journals being automatically indexed in April 2022.

### 4.2. What lies ahead

The NLM Medical Text Indexer (MTI) continues to be improved and expanded using the latest technology. The next generation of MTI (MTIX) has been developed from the beginning as a Machine Learning/Deep Learning program, presented by Rae et al. (2021) at the 2021 BioASQ workshop. MTIX is being developed in the National Center for Biotechnology Information (NCBI) division of NLM where it will be integrated directly into the PubMed Data Management System (PMDM; Gollner and Canese, 2017). This direct integration into the PMDM data flow will eventually allow new articles uploaded from a publisher to be immediately automatically indexed before the article even shows up in PubMed. There are several efforts continuing to be worked on in the MEDLINE 2022 project (NLM, 2021), including expanding the identification of genes, proteins, and chemicals. The move from the existing MTIA to the new MTIX is planned for Fall 2023.

Despite the important advancements achieved in semantic indexing so far, there is still a lot of room for improvement in terms of specific challenges still persisting in the field. For example, new MeSH descriptors, introduced during the extension of the MeSH thesaurus through annual updates (Nentidis et al., 2021), are not covered by training datasets developed based on previous annotations. As a result, state-of-the-art systems for semantic indexing, relying on supervised machine learning, can not handle these specific labels. These include emerging descriptors, representing topics that were previously not present in the literature, such as the COVID-19 pandemic, or new descriptors introduced to update the current views and priorities in the biomedical domain, as regards the indexing of the literature.

The new MTIX system has been designed to be more adaptive so that it can easily handle new journals, new MeSH terms, and any MeSH term drift that might occur. MTIX uses the journal descriptors (MeSH terms that describe what a journal is about, e.g., Veterinary Medicine) to train groups of like journals, so new journals will by default be indexed based on their journal descriptor training group until the next time MTIX is fully retrained. Both new MeSH terms and any MeSH term that has drifted from its meaning would be handled in the same way. NLM has established a team of experienced in-house curators that will manually index a small set of articles that involve new MeSH terms (or drifted terms) to provide MTIX with a set of validated indexed articles to train with. The new training sets will include both true positive occurrences of the new/drifted MeSH terms and any false positive examples the curators can identify, so that MTIX has a more balanced training set to work with. Experiments are ongoing to determine exactly how many examples of each new/drifted MeSH term are needed to ensure the best training for MTIX.

To ensure that MeSH indexing continues to be the quality product that it is, NLM has the team of experienced curators review and re-index specific types of articles and random samples of the automatic indexing. Any corrections made by the curators are fed back into improving MTIA and corrected on any affected articles. Journals where chemicals, genes, and proteins are more frequently found are targeted in this review due to MTIA not doing as well with them. The random sample of 100 (at time of writing) daily articles is chosen from any remaining articles from across the daily processing. This set of 100 articles statistically provides an adequate random sampling for daily review. NLM is continuing to evaluate this number to ensure that we cover the literature and will adjust as the results of these evaluations and user feedback dictate.

Still, the ever-expanding information needs of domain experts suggest that further extensions of semantic indexing would be useful in several directions, even beyond scientific literature and/or MeSH descriptors. This includes indexing other types of documents such as clinical trials, healthcare-project summaries, biomedical patents, and clinical reports, as well as indexing in other languages beyond English (Rodriguez-Penagos et al., 2020; Gasco et al., 2021; Miranda-Escalada et al., 2022). In addition, indexing with certain types of labels is another interesting direction, which may also require labels from different vocabularies such as the SNOMED-CT (Donnelly et al., 2006; Miranda-Escalada et al., 2022; Lima-López et al., 2023) or the UMLS (Bodenreider, 2004). In these directions, annotated data are usually scarce or missing, raising the need for novel methods, beyond supervised learning, such as weakly supervised, few-shot, and zero-shot learning for emerging or fine-grained descriptors (Mylonas et al., 2020; Nentidis et al., 2020).

## Data availability statement

The datasets presented in this study can be found in online repositories. The names of the repository/repositories and accession number(s) can be found at: http://participants-area.bioasq.org/datasets/, Training v.2022.

## Author contributions

AK and GP originated the BioASQ Challenge. AN was co-organizing the BioASQ challenge since 2017. JM provided the MTI tool for the challenge and has supported the BioASQ challenge since the beginning. AK, JM, and AN drafted the manuscript. All authors reviewed the manuscript. All authors contributed to the article and approved the submitted version.

## Footnotes

^1^ https://www.nlm.nih.gov/bsd/licensee/baselinestats.html

^2^PubMed Query: 1965:1971/02/06[dcom] AND MEDLINE [sb] returns 1,000,339 (results June 27, 2022).

^3^ http://www.hubmed.org/

^4^ https://clustermed.info/

^5^ https://www.wolterskluwer.com/en/solutions/ovid

^6^Frequently Asked Question about Indexing for MEDLINE (website): https://www.nlm.nih.gov/bsd/indexfaq.html.
